# Trained immunity: An unexplored component of IBD pathogenesis

**DOI:** 10.1093/ibd/izag119

**Published:** 2026-07-17

**Authors:** Michael Doulberis, Stergios A Polyzos, Abbas Yadegar, Jannis Kountouras

**Affiliations:** Department of Gastroenterology and Hepatology, University Hospital Zurich, University of Zurich, Zurich, 8091, Switzerland; Division of Gastroenterology and Hepatology, Medical University Department, Kantonsspital Aarau, Aarau, 5001, Switzerland; Department of Medicine, Second Medical Clinic, Ippokration Hospital, Aristotle University of Thessaloniki, Thessaloniki, 54642, Macedonia, Greece; Department of Medicine, Second Medical Clinic, Ippokration Hospital, Aristotle University of Thessaloniki, Thessaloniki, 54642, Macedonia, Greece; First Laboratory of Pharmacology, School of Medicine, Aristotle University of Thessaloniki, Thessaloniki, 54642, Macedonia, Greece; Foodborne and Waterborne Diseases Research Center, Research Institute for Gastroenterology and Liver Diseases, Shahid Beheshti University of Medical Sciences, Tehran, Iran; Department of Medicine, Second Medical Clinic, Ippokration Hospital, Aristotle University of Thessaloniki, Thessaloniki, 54642, Macedonia, Greece

**Keywords:** IBD, inflammatory bowel disease, trained immunity, innate immunity, ulcerative colitis, Crohn’s disease

## Abstract

Trained immunity (TI) describes the capability of the innate immune system and its tissue-resident cells to acquire long-lasting functional adaptations following an initial inflammatory or microbial insult. These changes are maintained through coordinated immunometabolic rewiring and epigenetic remodeling, resulting in amplified or altered responses upon subsequent challenges. Inflammatory bowel disease (IBD) is a chronic, relapsing disorder of the gastrointestinal tract, and TI offers a useful framework for understanding disease persistence, relapse proneness, and incomplete resolution despite effective targeting of adaptive immune pathways. While TI has been extensively studies in various chronic inflammatory disorders, its role in IBD remains rather underexplored and largely confined to early preclinical evidence. Within this narrative review, we aim to address this gap by providing current insights into the potential pathogenetic mechanisms underpinning TI in IBD. We examine how the shaping of the behavior of monocytes, macrophages, and their bone marrow progenitors and how steady exposure to microbial ligands, dysbiotic metabolites, and metabolic stressors within the intestinal lumen may establish a sustained proinflammatory “memory” in the context of IBD. From a translational perspective, we consider how TI may favor relapse even during clinical remission and discuss its potential relevance for patient stratification, biomarker identification, and the development of novel therapeutic strategies. Ultimately, integration of TI into IBD pathophysiology may corroborate more lasting, mechanism-based approaches to remission.

## Graphical abstract

**Graphical Abstract:**
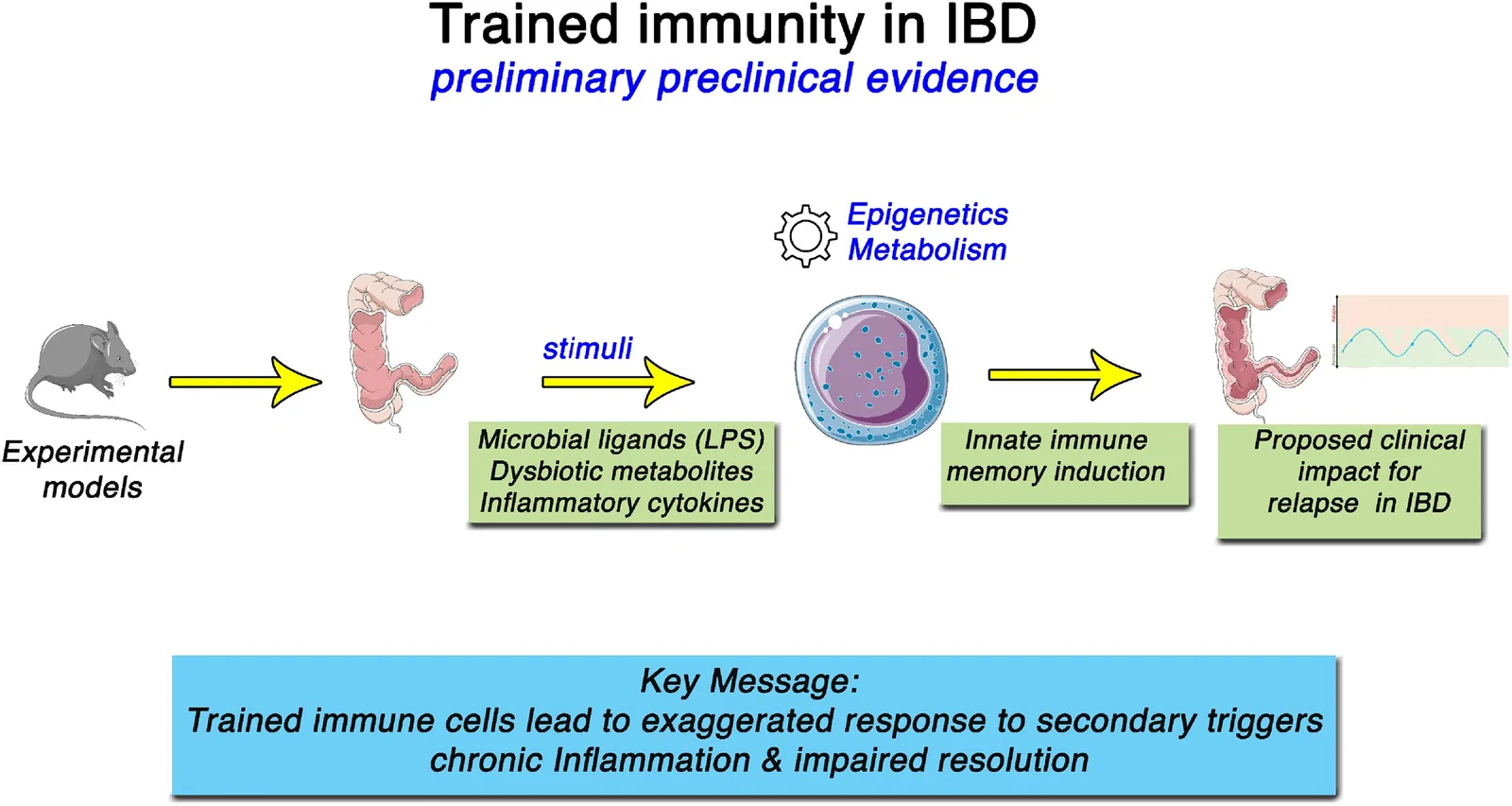

## Introduction

Trained (innate) immunity (TI) represents a novel concept in modern immunology that describes the ability of innate immune cells to acquire a memory-like phenotype after an initial challenge. Contrary to adaptive immunity, which relies mostly on antigen-specific lymphocytes, TI is mediated by both epigenetic and metabolic reprogramming of a plethora of immune cells including monocytes, macrophages, natural killer cells, and even nonimmune cells such as endothelial and epithelial cells. This reprogramming confers a long-lasting, recallable hyperresponsive state, enabling the host to mount an enhanced innate response upon later stimulation, even in the absence of antigen specificity.^1^^,^^2^ Key conceptual differences between TI, innate immune tolerance, and adaptive immune memory are enclosed in the accompanying Table 1.

**Table 1:** Levels of TI: Cellular compartments, metabolic–epigenetic programs, and relevance to IBD.

Level of trained immunity	Cell type	Trigger(s)	Dominant metabolic pathways	Key epigenetic marks	Relevance to IBD
**Peripheral TI**	Circulating monocytes	β-glucan, BCG, LPS, oxLDL, microbial ligands	↑ Glycolysis (mTOR–HIF-1α), glutaminolysis, mevalonate pathway	H3K4me3, H3K27ac, H3K18la/H3K27la	Enhanced cytokine production, exaggerated responses upon microbial re-exposure
**Peripheral TI**	Tissue macrophages	Dysbiotic microbiota, DAMPs, metabolic stress	Glycolysis, altered TCA cycle, fumarate accumulation	Reduced H3K9me3, increased activating histone marks	Sustained mucosal inflammation, impaired resolution
**Central TI**	Bone marrow progenitors (HSPCs)	Microbial translocation, systemic inflammation	Metabolic rewiring of progenitors (glycolysis, cholesterol metabolism)	Persistent chromatin accessibility at inflammatory loci	Long-term bias toward proinflammatory myelopoiesis, relapse proneness
**Tissue TI**	Intestinal macrophages	Continuous microbial exposure, cytokine milieu	Aerobic glycolysis, mevalonate pathway	H3K4me3, H3K27ac	Barrier dysfunction, chronic inflammatory tone for IBD, albeit not proven
**Epithelial inflammatory memory**	Intestinal epithelial stem/progenitor cells	Inflammatory cytokines, microbial signals	Metabolic stress–linked pathways	Stable chromatin accessibility changes	Altered barrier repair, increased susceptibility to recurrent inflammation

Abbreviations: BCG, Bacillus Calmette–Guérin; DAMP, damage-associated molecular pattern; HIF-1α, hypoxia-inducible factor-1 alpha; HSPC, hematopoietic stem and progenitor cell; IBD, inflammatory bowel disease; LPS, lipopolysaccharide; oxLDL, oxidized low-density lipoprotein; TCA, tricarboxylic acid cycle; TI, trained immunity.

TI is initiated when pathogen-associated molecular patterns, such as β-glucan or Bacillus Calmette–Guérin, engage pattern recognition receptors expressed on the surface or within the cytoplasm of innate immune cells. This interaction triggers alterations in intracellular signaling pathways and cellular metabolism, ultimately resulting in sustained metabolic and epigenetic reprogramming. Consequently, innate immune cells become “primed,” enabling enhanced production of proinflammatory cytokines—including tumor necrosis factor α (TNF-α), interleukin (IL)-6, and IL-1β—as well as chemokines such as C-X-C motif chemokine ligands CXCL9 to CXCL11 upon subsequent stimulation. Such persistent functional reprogramming, although protective in acute infections, may turn to maladaptive under chronic inflammatory circumstances. Crucially, although TI is a well-established contributor to a wide range of chronic inflammatory disorders, its role in inflammatory bowel disease (IBD) remains largely unexplored. Emerging preclinical data point to a possible involvement of trained innate immune responses in both the onset and recurrence of IBD; however, direct evidence in human Crohn’s disease (CD) and ulcerative colitis (UC) is still lacking.^3–5^

This review aims therefore to address this gap by critically evaluating the potential molecular mechanisms underpinning TI in the context of IBD. While TI provides a compelling conceptual framework for understanding IBD pathogenesis, current insights are predominantly derived from early-stage animal studies, underscoring the urgent need for well-designed translational research in human populations.

## Molecular drivers of TI: An immunometabolic–epigenetic axis

At the molecular level, the induction of TI is driven by the convergence of metabolic and epigenetic reprogramming, which together sustain a long-lasting proinflammatory phenotype. While these mechanisms are well established in experimental models of innate immune memory, their precise contribution to IBD pathogenesis remains largely hypothetical.^6–8^

A defining feature of TI is the metabolic shift toward aerobic glycolysis (the “Warburg effect”), implicating the mTOR/HIF-1α pathway. Within the IBD context, this pathway is particularly relevant: the inflamed intestinal epithelium is characterized by severe hypoxia, a condition that stabilizes HIF-1α.^9^ This hypoxic microenvironment may promote the polarization of infiltrating lamina propria myeloid cells toward a glycolytic phenotype, resulting in lactate accumulation and enhanced chromatin accessibility through histone lactylation. Nevertheless, whether this specific metabolic–epigenetic axis operates in the lamina propria of patients with CD and/or UC remains to be directly demonstrated.^6–8^

Metabolic reprogramming further acts upstream to reinforce the persistence of key epigenetic modifications, particularly H3K4me3 at gene promoters and H3K27ac at enhancers of proinflammatory genes such as *TNFA* and *IL6*. Such epigenetic marks maintain innate immune cells in a “primed” state, enabling enhanced and rapid cytokine production upon subsequent microbial stimulation.^9–11^ Consistent with this, increased H3K27ac levels in β-glucan–trained monocytes have been associated with reduced expression of the NAD^+^‐dependent class III histone deacetylase Sirtuin‐1.^9^

In addition to activating marks, repressive histone modifications are dynamically regulated during TI. For example, levels of the repressive mark H3K9me3 are reduced at promoters of proinflammatory genes following β-glucan–induced training, facilitating augmented transcriptional activation.^11^

Similarly, modulation of H3K27me3 has been observed in β-glucan models. Pharmacological inhibition of KDM6 demethylases using GSK-J4 attenuates lipopolysaccharide-induced cytokine production in human macrophages, although its precise role in TI remains incompletely defined.

**Figure 1:**
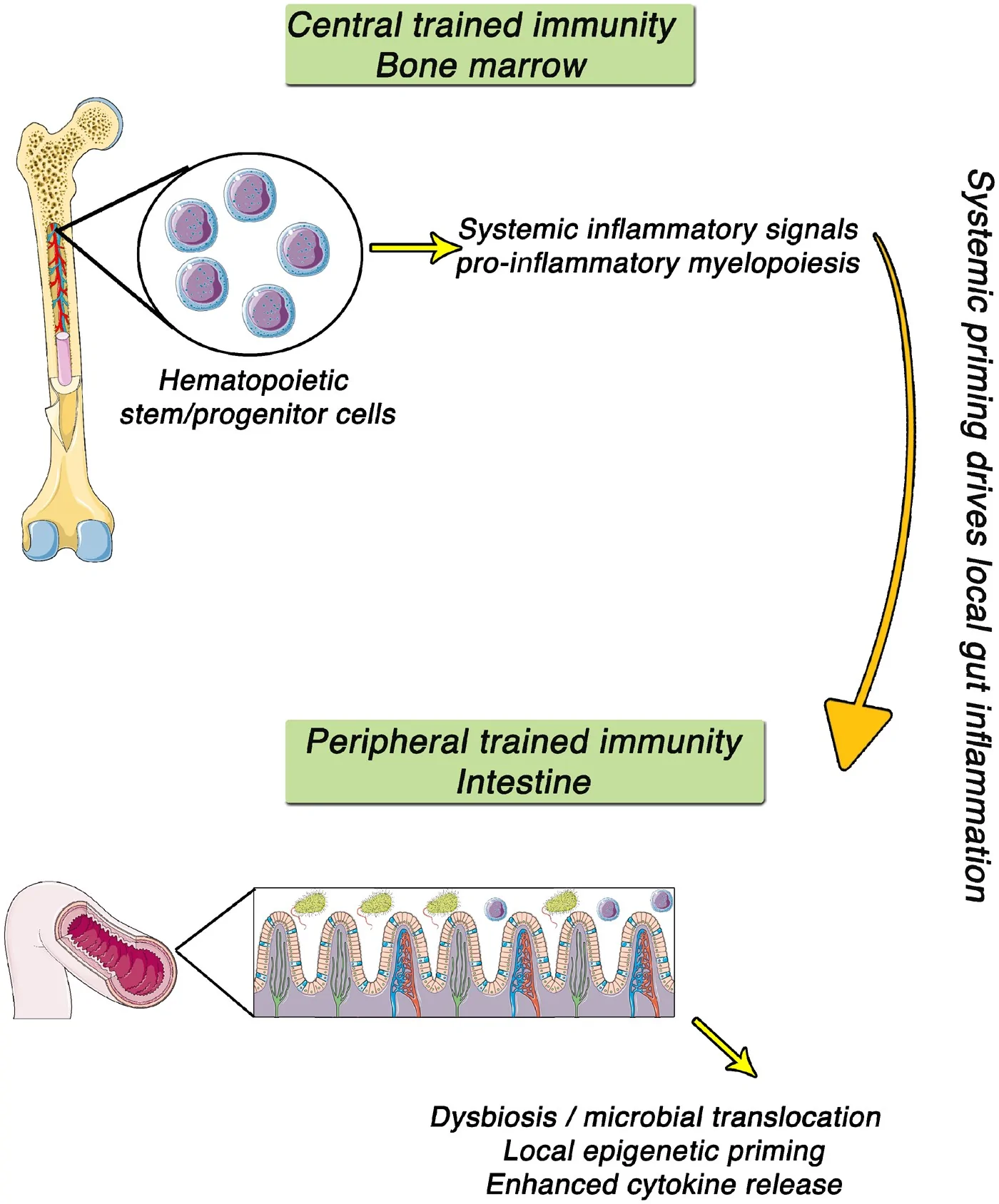
Central and peripheral trained immunity in the pathogenesis of inflammatory bowel disease (IBD). The schematic depicts the dual-compartment model of trained immunity contributing to chronic intestinal inflammation. **For central trained immunity,** systemic inflammatory signals and microbial translocation induce metabolic rewiring (eg, glycolysis, mevalonate pathway) and epigenetic reprogramming in bone marrow hematopoietic stem and progenitor cells. This results in a long-term bias toward proinflammatory myelopoiesis, increasing the pool of “primed” circulating monocytes. **For peripheral trained immunity, t**hese primed monocytes infiltrate the intestinal tissue, where they are further exposed to dysbiotic microbial ligands and inflammatory cues. This leads to sustained metabolic–epigenetic remodeling (eg, H3K4me3, H3K27ac) in tissue-resident macrophages, resulting in an exaggerated inflammatory response, impaired resolution, and persistent barrier dysfunction, which collectively drive IBD chronicity and relapse.

Overall, TI reflects the integration of metabolic rewiring, epigenetic remodeling, and sustained functional reprogramming, as summarized in Figure 1. Although emerging transcriptomic data from chronic inflammatory diseases lend support to this framework, translational evidence in human IBD is still limited. Importantly, direct proof that these epigenetic alterations drive disease relapse in patients is lacking, underscoring the need for longitudinal clinical studies in IBD.

## Peripheral vs central TI

TI operates along two interconnected axes: a peripheral and a central pathway. The peripheral axis was initially described in circulating monocytes that undergo functional reprogramming, but it has since been expanded to include additional cell types such as tissue-resident macrophages and epithelial cells within intestinal mucosa. In contrast, the central axis involves the epigenetic reconfiguration of hematopoietic stem cells within the bone marrow. This mechanism provides a compelling explanation for the durability of innate immune memory, as bone marrow–derived “pretrained” myeloid cells are continuously supplied to peripheral tissues, maintaining a heightened state of responsiveness even after the initial stimulus has resolved.^12^

Of note, a “gut-bone marrow axis” has recently been described in IBD.^13^ In this context, signals derived from intestinal dysbiosis—particularly type I interferon pathways—can induce maladaptive epigenetic imprinting in bone marrow granulocyte-monocyte progenitors. This central reprogramming drives the persistent generation and recruitment of “pretrained,” hyperinflammatory myeloid cells to the intestinal mucosa, thereby contributing to the relapsing course of the disease.^13^

While TI likely evolved as a protective mechanism to enhance rapid pathogen clearance, its sustained or dysregulated activation is increasingly recognized as a contributor to chronic inflammatory disorders. Emerging evidence suggests that TI may influence disease onset, severity, and relapse risk, while also modulating responses to therapy.^6–8^ Within the intestinal microenvironment, continuous exposure to microbial and metabolic signals—often linked to dysbiosis—can prime gut macrophages and innate lymphoid cells toward a TI phenotype. This shift may compromise epithelial barrier integrity, disrupt immune tolerance, and lower the threshold for inflammation.^9^^,^^10^ Moreοver, this maladaptive innate memory may attenuate mucosal healing, reinforcing the self-perpetuating, relapsing-remitting cycle nature of IBD.^9^^,^^10^ As summarized in the accompanying Table 1, a deeper understanding of these TI axes is essential for clarifying their role in the pathogenesis of CD and UC.

## Trained immunity and IBD pathogenesis

**Figure 2:**
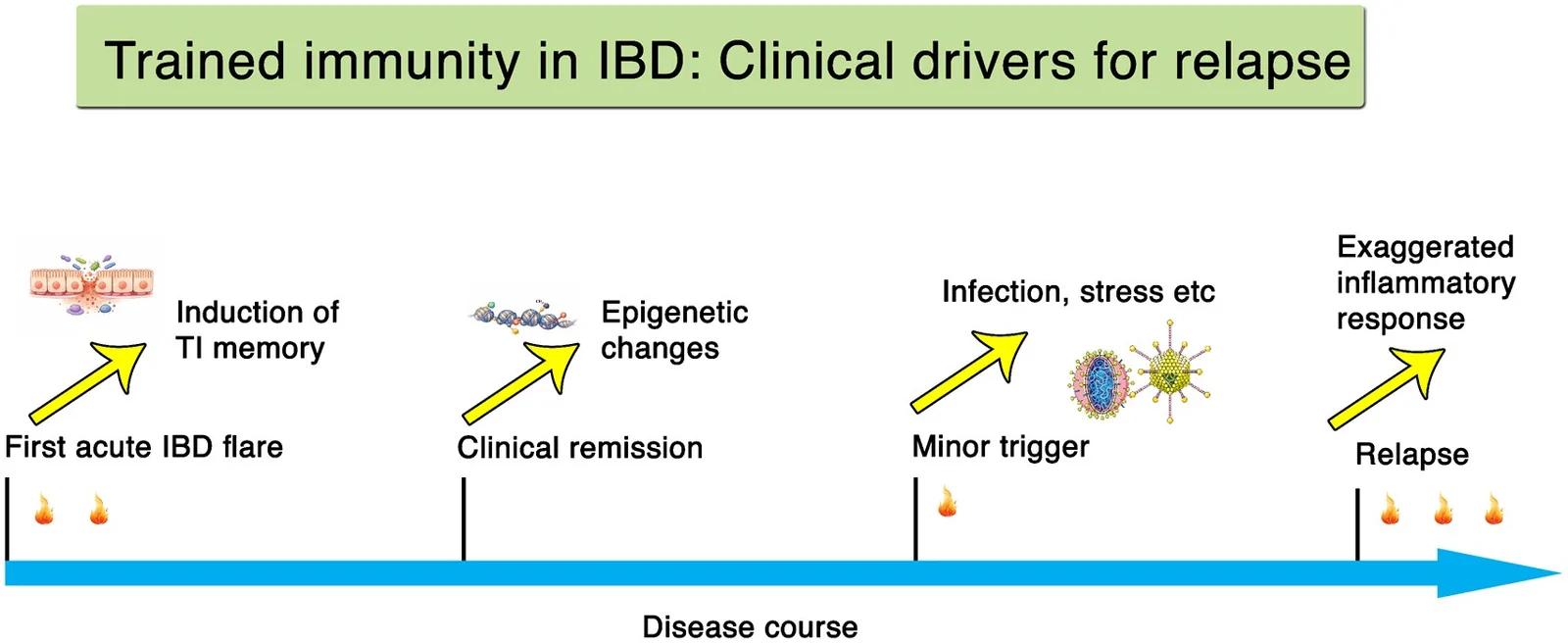
Trained immunity (TI) as a driver of chronic intestinal inflammation and relapse in inflammatory bowel disease (IBD). The timeline illustrates the hypothesis that TI functions as a mechanism underlying relapse in IBD. First, in **acute IBD flare, i**nitial exposure to microbial ligands and inflammatory cues induces metabolic and epigenetic reprogramming in myeloid cells. Second, in c**linical remission, d**espite apparent clinical and endoscopic resolution, persistent “molecular memory” is maintained through epigenetic priming (eg, histone modifications). Third, in a m**inor trigger,** environmental or gut-associated stressors act as secondary signals, interacting with primed cells. Fourth, in **relapse,** primed myeloid cells respond with an exaggerated proinflammatory cytokine burst, leading to disease reactivation and chronicity.

Recent research on TI in gastrointestinal diseases has primarily centered on intestinal inflammation. The cellular and systemic integration of TI in IBD, from microbial and metabolic triggers to perpetual innate immune activation and disease relapse, is illustrated in Figure 2.

Under inflammatory conditions, TI may contribute to the worsening of intestinal pathology. Mincle (macrophage-inducible C-type lectin) (encoded by the *Clec4e* gene) is a pattern recognition receptor predominantly expressed by myeloid cells that detects both endogenous danger signals and translocated microbial ligands following disruption of the intestinal barrier. Activation of this pathway promotes communication along the gut–bone marrow axis, inducing metabolic and epigenetic reprogramming of bone marrow granulocyte–monocyte progenitors and thereby establishing central TI. Importantly, Mincle-deficient (Mincle^−/−^) mice, which lack this innate sensing pathway, are protected from such systemic inflammatory imprinting. In models of dextran sulfate sodium–induced colitis, these mice display markedly reduced generation of pretrained, hyperinflammatory myeloid populations, resulting in attenuated disease severity and pathological manifestations. Collectively, these findings demonstrate that Mincle signaling is required for myeloid progenitor reprogramming and identify Mincle-mediated TI as a central driver of intestinal inflammation and IBD pathogenesis.^14^

IBD is characterized by chronic, relapsing inflammation of the gastrointestinal tract.^15^^,^^16^ Although the etiology is multifactorial, disruption of innate immune regulation is increasingly recognized as a central driver of disease onset and progression.^17^ TI may serve as a key link between environmental triggers and sustained innate immune dysregulation in IBD. TI-triggered macrophage and monocyte dysregulation in IBD contributes to epithelial barrier dysfunction (permeability and cytokine), chemokine overproduction, and impaired tolerance to commensal pathogens.^18^

In this context, reprogramming of innate immune cells may exert either protective or pathogenic effects in infectious, inflammatory, and autoimmune diseases.^4^ In both forms of IBD, the immune system aberrantly targets components of the gastrointestinal tract. Genes positioned near genome-wide association study–defined susceptibility loci for CD and UC were significantly overrepresented among the top differentially expressed genes in monocytes with enhanced inflammatory activity and in the core monocyte cluster subpopulations. These populations correspond to distinct inflammatory mononuclear phagocyte subsets identified by single-cell RNA sequencing of the colonic lamina propria.^19^ Notably, enhanced inflammatory activity–related transcriptional signatures were markedly increased in both inflamed and noninflamed intestinal tissues relative to tissues from healthy controls.^19^^,^^20^

Moreover, several genetic susceptibility loci linked to IBD are also involved in regulating innate immune responses and epigenetic mechanisms associated with TI. IBD pathogenesis involves over 200 genetic risk loci, many of which encode proteins essential for innate immunity, microbial recognition, and autophagy, including NOD2 (nucleotide-binding oligomerization domain-containing protein 2).^21^ Polymorphisms in NOD2, an innate immune sensor of bacterial peptidoglycans, are not only linked to defective microbial clearance, but also orchestrate TI responses,^22^ highlighting a central role for NOD2 in the induction of TI.

NOD2-mediated immune tolerance is particularly relevant to CD, which is marked by dysregulated immune homeostasis and excessive inflammatory responses to intestinal microbiota.^23^

The capacity of NOD2 to function across both innate and adaptive immune signaling pathways enables it to drive either TI or immune tolerance via distinct mechanisms. This functional duality positions NOD2 as an attractive therapeutic target in inflammatory and infectious diseases, including IBD. Nevertheless, the outcome of NOD2-mediated immune regulation is shaped by genetic background and the surrounding immune microenvironment. Therefore, further comprehensive investigations are needed to elucidate its complex regulatory networks and to provide a robust theoretical framework for the development of NOD2-targeted therapeutic strategies.

Perturbations in macrophage polarization, including dysregulation of the M1/M2 axis, have been linked to aberrant inflammatory responses in IBD.^24^^,^^25^ In this respect, a recent preclinical study using a dextran sulfate sodium–induced murine model^26^ reported that wheat fiber supplementation confers protection against colitis. Notably, this protective effect appeared to be independent of short-chain fatty acids (SCFAs) and was linked to the preservation of gut microbiota diversity, including the sustained presence of *Bacteroides thetaiotaomicron*. Interestingly, its presence in gnotobiotic mice resulted in fecal metabolites that reprogrammed macrophages toward an M2-like phenotype.^26^

Bone marrow reprogramming may also perpetuate intestinal inflammation. Trained phenotypes induced in progenitor cells have been observed in murine colitis models, resulting in long-lasting biases toward inflammatory monocyte lineages.^27^

Consistent with these findings, Kuang et al^28^ showed that UC can aggravate anatomically distant inflammatory conditions such as periodontitis through priming bone marrow in order to ramp up myelopoiesis. This highlights the fueling of mucosal inflammation toward systemic disease and its potential link to trained hematopoietic responses.^28^

Both periodontitis and IBD are chronic inflammatory disorders characterized by immune dysregulation and microbial imbalance. The oral cavity functions as a key reservoir for pathogens, including *Porphyromonas gingivalis* and *Helicobacter pylori*, which contribute to systemic inflammation through the oral–gut axis.^29^^,^^30^ These microorganisms employ similar immune evasion strategies and proteolytic activities that facilitate persistent colonization and tissue damage.^31^ In particular, *P gingivalis* has been implicated in the pathogenesis of IBD. Emerging evidence suggests that targeting its virulence factors—such as through gingipain inhibition (eg, KYT-36)—or restoring microbial balance using probiotics like *Lactobacillus rhamnosus* GG may alleviate colitis, supporting a conceptual framework for integrated, cross-organ management of inflammatory diseases.^32^^,^^33^

Importantly, these pathogens may also act as drivers of TI. *H pylori* promotes chronic disease persistence by inducing trained responses,^34–36^ whereas *P gingivalis*–derived lipopolysaccharide triggers metabolic and epigenetic reprogramming of fibroblasts (gingival, intestinal) via the PI3K/AKT pathway.^37^ Collectively, these findings suggest that fibroblast-mediated TI may represent a shared mechanistic pathway underlying the recurrence of both IBD and periodontitis. Thus, the interplay between oral dysbiosis and gut immune responses emerges as a critical, yet underexplored, contributor to IBD recurrence, highlighting the need for microbiome-targeted strategies that address innate memory across distinct anatomical niches.

Additionally, recent transcriptomic and epigenetic analyses of monocytes from IBD patients have confirmed enrichment in histone marks linked to TI (eg, H3K4me3, H3K27ac), even during remission phases. These modifications may underlie the exaggerated cytokine responses observed upon microbial re-exposure.^13^ Such findings support the hypothesis that persistent TI contributes to flare-prone mucosal environments in IBD.

In murine colitis models, dietary succinate supplementation has been shown to alleviate intestinal inflammation via modulation of the IL-4Rα/HIF-1α axis, further supporting the therapeutic relevance of immunometabolic interventions.^38^ Consequently, the metabolic status of the intestinal microenvironment dictates the longevity of innate immune memory, potentially rendering these cells refractory to conventional anti-inflammatory therapies.

Moreover, the gut microbiota plays a central role in shaping TI dynamics, especially in the context of IBD. Dysbiosis—a hallmark of IBD—alters the production of key microbial metabolites such as SCFAs, bile acids, and tryptophan catabolites.^39^

Zelante et al^40^ demonstrated that microbial tryptophan catabolites act as ligands for the aryl hydrocarbon receptor (AhR), promoting IL-22 production and maintaining epithelial barrier function—a mechanism that may be disrupted in dysbiosis-driven IBD. Likewise, microbial tryptophan metabolism produces AhR ligands that regulate mucosal immunity and barrier integrity, potentially skewing the immune balance toward tolerance or activation depending on the ligand availability and local immune state.^40^

Together, these findings support a model in which microbiota-derived signals modulate TI dynamics in preclinical IBD, influencing the transition from acute immune activation to chronic inflammation. This interplay might present opportunities for therapeutic targeting of innate immune memory through epigenetic or metabolic reprogramming.

## Possible therapeutic and clinical implications

Table 2 synthesizes candidate therapeutic strategies that may modulate ΤΙ in the context of IBD, grouped by mechanistic comments and level of evidence. Statins, which block HMG-CoA reductase, the rate-limiting enzyme of the mevalonate pathway, have been reported in in vitro studies to abrogate the induction of TI by hampering the synthesis of key intermediates required for histone modifications.^10^ Likewise, metformin, an AMPK activator and mTOR inhibitor, commonly used for type 2 diabetes mellitus, counteracts glycolytic flux and has been displayed to decrease inflammation and innate cytokine hypersecretion in murine colitis models, proposing therefore a potential as a possible adjunctive maintenance therapy in the context of IBD.^9^

**Table 2:** Clinical and translational implications of TI in IBD.

Clinical context in IBD	TI-related interpretation	Observable clinical pattern	Implications for clinical monitoring	Therapeutic considerations
**Clinical remission with recurrent flares**	Persistence of innate immune memory despite apparent resolution of inflammation	Flare-prone remission, frequent relapses after treatment tapering	Need for closer follow-up despite clinical remission	Cautious de-escalation; consideration of maintenance optimization
**Steroid-responsive but steroid-dependent disease**	Amplified innate immune responsiveness sustaining inflammatory tone	Rapid symptom recurrence after steroid withdrawal	Repeated discordance between short-term response and long-term control	Early transition to steroid-sparing strategies
**Postoperative Crohn’s disease**	Residual trained immune responses after removal of inflamed bowel segments	Early endoscopic or clinical recurrence	Early postoperative surveillance	Consideration of early prophylactic medical therapy
**EIMs**	Systemic dissemination of trained innate immune responses	Parallel intestinal and extraintestinal inflammatory activity	Integrated assessment beyond intestinal symptoms	Preference for systemic rather than gut-restricted therapies
**Chronic dysbiosis or persistent microbial exposure**	Recurrent stimulation of innate immune training	Incomplete or unstable remission	Attention to microbial and environmental modifiers	Adjunctive strategies addressing microbial triggers
**Biomarker–symptom dissociation**	Sustained innate immune activation independent of clinical indices	Elevated inflammatory markers with mild symptoms or vice versa	Combined clinical and biomarker-based monitoring	Avoidance of decisions based on single disease activity parameters

Abbreviations: EIM, extraintestinal manifestation; IBD, inflammatory bowel disease; TI, trained immunity.

TI remains perpetual by lasting epigenetic marks, particularly histone modifications such as H3K4me3 and H3K27ac at proinflammatory gene loci. Certain therapeutic strategies that selectively target to reverse these epigenetic signatures—termed “de-training”—are gaining recently worldwide attention. Histone deacetylase inhibitors and histone methyltransferase inhibitors have demonstrated the potential to restore chromatin accessibility and attenuate excessive cytokine overresponses. Nanomedicine may offer a more attractive and targeted approach: engineered nanobiologics that selectively deliver metabolic or epigenetic inhibitors (eg, itaconate derivatives) to CD68+ macrophages in the inflamed intestine appear promising to suppress maladaptive training while preserving systemic immune competence.^41–43^

Itaconate itself, a shunt product of tricarboxylic acid cycle, has emerged as a key player of TI. Through activation of the Nrf2 pathway, itaconate curtails inflammatory gene expression and orchestrates resolution phase of inflammation. These insights underscore the therapeutic potential of modulating the metabolic–epigenetic axis that defines ΤΙ responses in IBD.^41^

A further major issue represents the IBD patients and their risk stratification: not all individuals with IBD exhibit the same immune profile, and only a subset may be driven primarily by TI mechanisms. Standard clinical metrics such as C-reactive protein or fecal calprotectin lack the granular resolution to distinguish between acute inflammatory flares and persistent, low-grade innate immune memory. Thus, the search for high-fidelity biomarkers is a priority to be addressed. Recent leaps in single-cell transcriptomic analyses have started unraveling the existence of distinct TI programs across monocyte populations, emphasizing interindividual and intracellular heterogeneity in distinct TI responses.^20^ Ex vivo monocyte stimulation assays, in which cells are rechallenged with microbial ligands to assess cytokine output (eg, TNF-α/IL-10 ratio), offer a potential diagnostic approach. Moreover, next-generation sequencing techniques now allow profiling of epigenetic landscapes—such as H3K4me3 enrichment at TNFA or IL6 promoters—which could offer a prognostic window into the risk stratification between relapse or therapeutic response).^11^^,^^42^^,^^43^ Additionally, recent single-cell expression quantitative trait loci analyses have uncovered immune-response genetic signatures across disease contexts, providing valuable insights for tailoring individualized therapies in IBD.^44^

The gut microbiome not only lies central to mucosal immunity, but also plays a critical role in shaping TI responses. Imbalance of the pathogen equilibrium (commonly referred as dysbiosis) in the context of IBD modifies the pool of microbial ligands and metabolites that engage innate immune sensors, such as Toll-like receptors and NOD-like receptors. Microbiota-derived SCFAs, for instance butyrate and propionate, upregulate histone acetylation and promote anti-inflammatory gene expression in monocytes and dendritic cells.^45^ Tryptophan catabolites, acting through the AhR, shape mucosal homeostasis, and their dysregulation synergizes to innate immune imbalance.^40^

These microbial signals not only impact local immune activation, but also may render bone marrow reprogramming, a phenomenon observed in murine models of colitis, in which progenitor cells acquire an inflammatory bias much later than the initial insult. This phenomenon helps us perceive the chronicity and recurrence of IBD flares.^14^^,^^45^^,^^46^ Such systemic effects may be mediated in part by bacterial translocation, which has been reported to promote TI in hematopoietic cells.^47^ Modulating the gut microbiota, whether through dietary intervention, prebiotics, or targeted probiotics capable of modulating TI pathways, offers an intriguing, though complex, treatment potential. For instance, select bacterial strains may “re-educate” innate immune cells by producing metabolites that antagonize trained inflammation.^14^^,^^45^^,^^46^ In this context, even microbial-derived immunomodulators such as cholera toxin have been displayed to reshape colonic immune responses, promoting IL-10 production and Treg (FoxP3+) polarization while suppressing inflammation-driven tumorigenesis in murine colitis–associated cancer models.^48^

Beyond preclinical findings, this approach has also been evaluated in clinical settings. In patients with CD experiencing active flare-ups, oral administration of the B subunit of cholera toxin resulted in a clinical response in approximately 40% of patients after 4 weeks, suggesting a promising strategy to “re-educate” dysregulated innate immune responses.^49^

Still, any attempt to interfere with TI must balance efficacy with safety. TI-modulating therapies may be best deployed as temporally limited interventions—“pulse therapies”—during transitions from active disease to remission, rather than continuous maintenance regimens.^4^

A novel and speculative avenue is the development of TI-based vaccines, such as Bacillus Calmette–Guérin or β-glucan–based formulations, which prime innate immune cells toward protective, rather than pathogenic, responses. Preclinical data in murine colitis models suggest such vaccines may prevent relapse and accelerate mucosal healing by establishing a regulatory trained phenotype in tissue-resident macrophages).^4^^,^^11^

Ultimately, reframing TI into the IBD narrative transforms the disease as a complex interplay between environmental cues, microbial ecosystems, and epigenetically rewired innate immune cells. While current pharmacological strategies are effective at alleviating symptoms, they frequently fall short of resolving the persistent, low-grade inflammation sustained by enduring innate immune memory. Recognizing TI as a “Janus-faced” mechanism—alluding to the ancient Roman god Janus, who is depicted with two faces looking in opposite directions—highlights its dual and contrasting roles: on one side, it serves as a beneficial, protective mechanism in host defense against infections; on the other, when persistently dysregulated, it becomes a harmful, maladaptive driver of chronic disease. This underscores the need to address the underlying epigenetic and metabolic reprogramming associated with TI, with the aim of resetting maladaptive immune responses at their source.

## Conclusion

Conventional therapies—and more recently, biologics—have significantly advanced the management of IBD. However, their limited ability to address underlying disease causality and prevent relapse highlights a persistent therapeutic ceiling. The integration of the TI paradigm suggests a necessary shift from broad, nonspecific immunosuppression toward targeted metabolic and epigenetic reprogramming. We envision a future in which true “immunological remission” is achieved through the resetting of hyperresponsive innate immune memory in tissue-resident cells and bone marrow progenitors. At present, however, the clinical application of this approach remains in its early stages. Longitudinal studies will be essential to determine whether profiling the TI-associated epigenetic landscape can serve as a reliable, high-fidelity biomarker for therapeutic stratification in IBD. By targeting the underlying inflammatory “memory,” rather than merely its downstream manifestations, this strategy may ultimately enable sustained, drug-free mucosal healing.

## Data Availability

No new data were generated or analysed in support of this research. This paper is based exclusively on previously published studies, which are cited within the article.
